# Comprehensive analysis of tobacco pollen transcriptome unveils common pathways in polar cell expansion and underlying heterochronic shift during spermatogenesis

**DOI:** 10.1186/1471-2229-12-24

**Published:** 2012-02-16

**Authors:** Said Hafidh, Katarína Breznenová, Petr Růžička, Jana Feciková, Věra Čapková, David Honys

**Affiliations:** 1 Laboratory of Pollen Biology, Institute of Experimental Botany, Academy of Sciences of the Czech Republic, Rozvojová 263, 165 02 Praha 6, Czech Republic; 2 Department of Plant Experimental Biology, Faculty of Science, Charles University in Prague, Viničná 5, 128 44 Praha 2, Czech Republic

## Abstract

**Background:**

Many flowering plants produce bicellular pollen. The two cells of the pollen grain are destined for separate fates in the male gametophyte, which provides a unique opportunity to study genetic interactions that govern guided single-cell polar expansion of the growing pollen tube and the coordinated control of germ cell division and sperm cell fate specification. We applied the Agilent 44 K tobacco gene chip to conduct the first transcriptomic analysis of the tobacco male gametophyte. In addition, we performed a comparative study of the Arabidopsis root-hair trichoblast transcriptome to evaluate genetic factors and common pathways involved in polarized cell-tip expansion.

**Results:**

Progression of pollen grains from freshly dehisced anthers to pollen tubes 4 h after germination is accompanied with > 5,161 (14.9%) gametophyte-specific expressed probes active in at least one of the developmental stages. In contrast, > 18,821 (54.4%) probes were preferentially expressed in the sporophyte. Our comparative approach identified a subset of 104 pollen tube-expressed genes that overlap with root-hair trichoblasts. Reverse genetic analysis of selected candidates demonstrated that Cu/Zn superoxide dismutase 1 (CSD1), a WD-40 containing protein (BP130384), and Replication factor C1 (NtRFC1) are among the central regulators of pollen-tube tip growth. Extension of our analysis beyond the second haploid mitosis enabled identification of an opposing-dynamic accumulation of core regulators of cell proliferation and cell fate determinants in accordance with the progression of the germ cell cycle.

**Conclusions:**

The current study provides a foundation to isolate conserved regulators of cell tip expansion and those that are unique for pollen tube growth to the female gametophyte. A transcriptomic data set is presented as a benchmark for future functional studies using developing pollen as a model. Our results demonstrated previously unknown functions of certain genes in pollen-tube tip growth. In addition, we highlighted the molecular dynamics of core cell-cycle regulators in the male gametophyte and postulated the first genetic model to account for the differential timing of spermatogenesis among angiosperms and its coordination with female gametogenesis.

## Background

Polarized extension of the pollen tube to deliver twin sperm cells to the female gametophyte is a fundamental process required for successful sexual reproduction and preservation of the floristic dominance of flowering plants. Understanding sexual reproduction is at the forefront of plant research and has benefited immensely from the development of genomic, transcriptomic and proteomics techniques in recent decades. Advances in microarray technology and development of gene chips for Arabidopsis, rice and other species have allowed studies on large-scale transcriptional profiling from diverse tissues, which has emerged as a key tool for identification of novel targets for functional genomics.

As in other angiosperms, the tobacco (*Nicotiana tabacum*) male gametophyte is formed within the anthers following meiotic division of the archesporial sporophytic cells to produce haploid microspores [1]. Thereafter, only two mitotic divisions occur. The first pollen mitosis (PMI) involves division of the haploid microspore to produce a gametic germ cell and a vegetative cell. These two cell types differ not only in the quantity and diversity of expressed transcripts [2,3], but also, crucially, in their fate. Whereas after pollen tube germination the germ cell undergoes a second mitotic division (PMII) to produce two male gametes (sperm cells), the vegetative cell adopts a different fate and acquires the critical task of ensuring growth of the pollen tube. At the stigma surface, the pollen tube germinates and penetrates the female stylar tissues to deliver the two sperm cells to the proximity of the egg and central cell for double fertilization. Among flowering plants, the production of sperm cells after pollen germination represents the ancestral pattern. However, over 30% of angiosperms, including *Arabidopsis thaliana*, complete the second division before pollen maturation [4,5]. No explanation has been proposed for the mechanisms that have imposed this regulon prompting differential timing in sperm cell proliferation in the male gametophyte of seed plants, nor have the evolutionary benefits of early gamete production been elucidated. Independent analysis of sperm cell DNA content revealed five patterns of sperm-cell development that differ with respect to relative timing of sperm-cell formation and maturation prior to fusion with the female gametes [5,6].

Pollen-tube germination represents a unique cellular phenomenon. The pollen tube grows through the female tissues in a polarized fashion similar to root-hair outgrowth, trichome specification, hyphal growth in fungi and extension of neuronal dendrites in the animal nervous system [7,8]. Thus, common factors are likely to be involved in the raw molecular cascades that initiate and control the single cell-tip expansion and morphogenesis, which are subsequently modulated by cell- or tissue-specific morphological demands. Studies using pollen tubes and root hairs as model systems have revealed a number of signaling molecules and regulatory pathways that promote and maintain the tip-growth characteristic. Recently, a study by Qin et al. [9] revealed a specific subset of genes that are induced in pollen tubes during growth through the pistil, but not when grown in vitro. Among this set of genes are potential receptor proteins that may respond to female cues for guidance of the pollen-tube tip growth. Collectively, these experiments demonstrated the direct dependence of cell-tip expansion on polarized exocytosis of vesicles at the apical growth region of the cell. Vesicular trafficking to the tip region is a structural requirement for tip growth and plays a significant role in feedback regulation of the tip-localized signaling to maintain the apical cell expansion [10].

Until recently, no major studies had analyzed the bicellular pollen transcriptome, with the sole exception of soybean (*Glycine max*) [11]. A first glimpse into the tobacco pollen transcriptome was reported by Xin and co-workers [12], who constructed an EST library from isolated tobacco sperm cells. This project aimed to identify proteins involved in sperm-egg recognition and fusion and identified over 1,864 EST sequences distributed in more than 1,050 clusters. This analysis discovered paternal candidate genes that could play a role during fertilization and in the early stages of embryogenesis. However, the sperm cell transcriptome alone represents only a small proportion of the total pollen-expressed transcripts and, although EST sequencing is sensitive, it provides only a limited list of expressed genes and thus minimizes comprehensive functional downstream analyses [3,12-14].

In the present study, we capitalized on the recent development of the Agilent 44 K tobacco microarray to present the first insight into tobacco pollen and pollen-tube transcriptomes, which represents a contrary experimental model from the tricellular pollen of Arabidopsis to a bicellular model of scientific, medical and commercial interest. In addition to broad-spectrum gene expression profiling, we performed a comparative analysis of the transcriptomes of tobacco pollen tubes and Arabidopsis root-hair trichoblasts [15-17] to uncover common pathways involved in polar cell expansion. To investigate their potential role in tobacco pollen-tube growth, we used gene ontology (GO) tools to allocate their biological roles, and applied an antisense-transfection approach to verify the function of selected candidate genes.

We further explored our data set to elucidate the control of male germ-cell proliferation and sperm-cell formation from a molecular perspective. In angiosperms, heterochronic alterations in cell cycle activity led to the developmental shift in the timing of germ-cell division and resulted in diversified patterns of spermatogenesis and gamete fusion during fertilization [4-6]. To elucidate the genetic network responsible for this shift among angiosperms, we investigated the expression of core cell-cycle repressors and activators at five stages of pollen development--namely, mature pollen and in vitro-germinated pollen tubes grown for 4, 13, 24 and 48 h - following completion of the germ cell mitotic division. On the basis of these results, we postulated a genetic model in which the male germ cell-cycle progression is coordinated with 'time to fertilization'.

The tobacco pollen and pollen-tube gene expression profiles generated in this study together with our analyses provide an ideal platform for future research into aspects of polarized cell expansion and control of spermatogenesis. In addition, cataloguing the expressed genes will provide an opportunity for development of gametophytic fluorescent markers with which cell behaviour and cell fate can be studied. The present study represents a step towards a comprehensive understanding of male gametogenesis, knowledge of which will provide an effective framework to improve current farming and breeding programs to sustain food requirements and for investigation of tobacco health implications.

## Results

### Application of the Agilent 44 k tobacco microarray to the *Nicotiana tabacum *male gametophyte transcriptome

**Figure 1:**
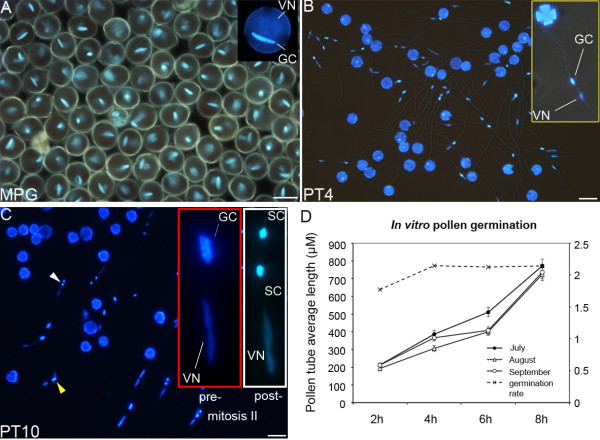
**Tobacco pollen and pollen tubes used for comparative transcriptomic analyses**. (**a**) Pollen from stage six of male gametophyte development [18], representing mature bicellular pollen with fully elongated germ cell (GC) alongside vegetative nucleus (VN), inset. Scale bar = 40 μM. (**b**) Pollen tubes cultivated in vitro for 4 h still at the bicellular stage. Scale bar = 60 μM. (**c**) Pollen tubes cultivated in vitro for 10 h with mixed population of premitotic germ cells (red inset) and those with newly formed sperm cells, SC (white inset). Scale bar = 60 μM. (**d**) Analysis of pollen-tube germination rate and length of pollen grains used in this study that were harvested at different times during the summer period. No significant differences in pollen fitness were observed, irrespective of time of collection.

In an effort to understand the diversity of gene expression accompanying bicellular tobacco pollen maturation and transcriptional changes with progamic phase induction, we applied tobacco microarray technology (imaGenes, Berlin, Germany) and performed comparative microarray analysis at two time points of male gametophyte development: mature pollen grains (MPG), and in vitro-germinated pollen tubes grown for 4 h (PT4). Tobacco mature pollen is bicellular when shed (Figure 1a), and is predicted to have a unique set of transcripts that are over- and under-represented in reference to phylogenetically advanced tricellular pollen. Pollen tubes grown for 4 to 48 h in vitro were used to verify the microarray expression by real-time quantitative RT-PCR (qRT-PCR) and to establish expression patterns of core cell-cycle regulators (Figure 1b, c). Two other tissue samples - leaves (L) and roots with substantial root-hair outgrowth (R) - were used as sporophytic references. The pollen grains were collected from tobacco plants grown in an outdoor greenhouse, so we investigated the possible effect of variable weather conditions on pollen-tube germination. Overall, no significant variation in pollen-tube germination rate or length throughout the harvesting period was observed (Figure 1d).

Normalized expression values (see Materials and Methods, Additional files 1 and 2: Figure S1a and Table S1) were used to perform cross-tissue comparisons for identification of preferentially expressed genes. To test the quality of the arrays, we stringently assessed our data sets to justify their reproducibility among replicates, and thus provide a feasible comparison between individual tissues. We used principal component analysis (PCA) and independently applied hierarchical clustering to visualize the relationship among the tissues as well as between biological replicates. The uniqueness of gametophyte versus sporophyte gene expression profiles was verified, together with the reproducibility between sample replicates (Additional file 1: Figure S1b). Scatterplots were used to visualize the distribution of *p*-values for individual probes relative to each other and in relation to the mean expression values. A close relationship between replicates and among tissues of similar origin was observed, whereas great variability in relation to disparate tissues was verified (Additional file 1: Figure S1c). Intensity-dependent-ratio (MvA) -derived values of array-array intensity independently supported the intra- and inter-relationships observed. Overall, the obtained normalized expression arrays were confirmed as being of good quality for downstream comparison.

**Figure 2:**
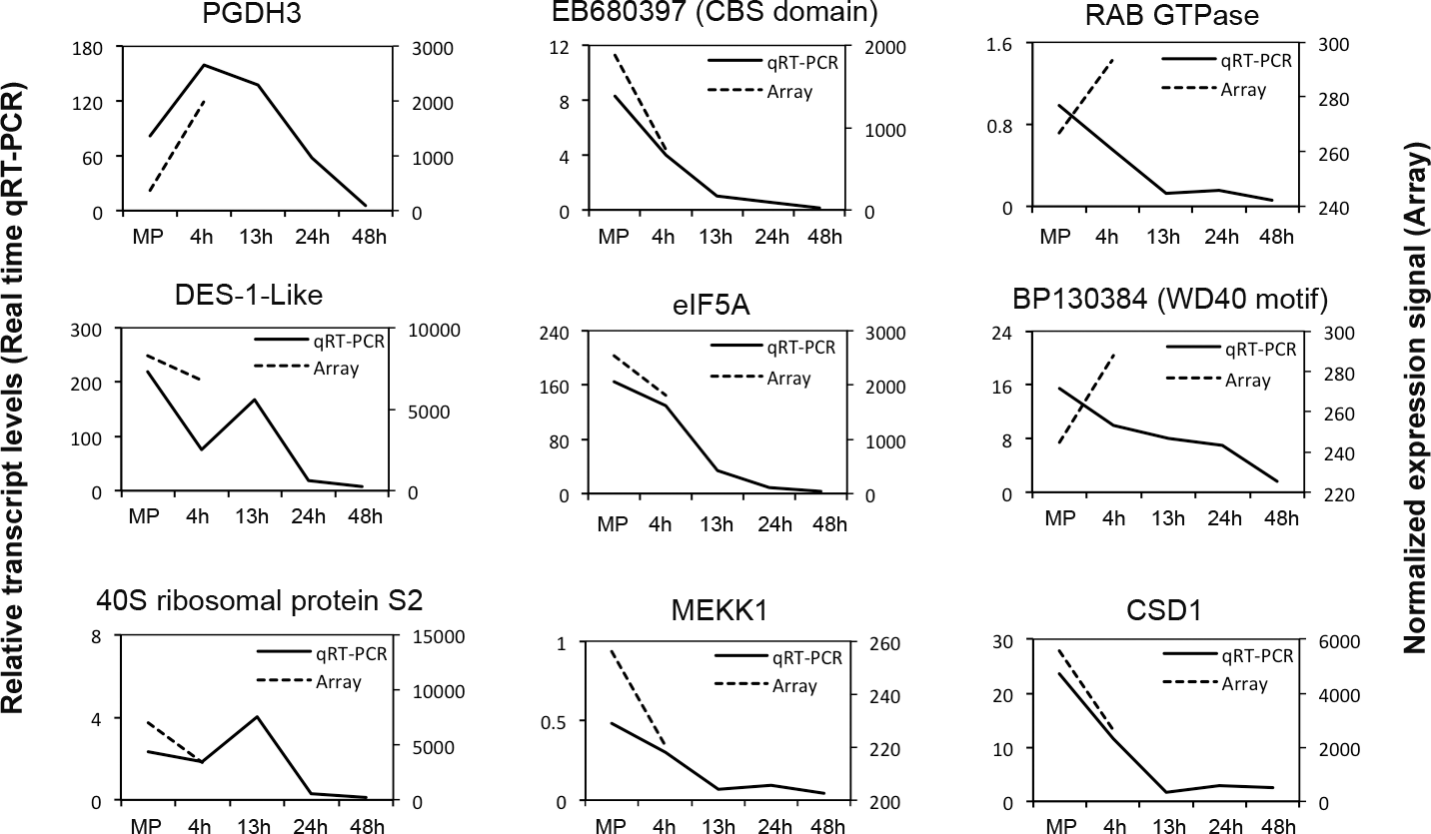
**Quantitative RT-PCR validation of microarray gene expression**. Relative expression levels of selected genes determined at mature pollen (MP) and at four stages of pollen-tube growth (4, 13, 24 and 48 h of cultivation). On the secondary axis, corresponding normalized expression signal at two pollen stages (MP and PT4) derived from microarray chip hybridization. The tobacco 18S RNA gene was used to normalize obtained Ct values to compute relative expression levels at each stage of the pollen gametophyte development.

We selected nine candidate genes to verify their microarray expression profiles by real-time qRT-PCR at the two developmental stages used for microarray analysis (MPG and PT4) and extended the analysis to three developmental stages post-PMII (13, 24 and 48 h after in vitro pollen germination). For all genes tested, with the exception of RAB GTPase and WD40 motif-containing protein (BP130384), qRT-PCR profiles corresponded to those of the microarray (Figure 2). The extended analysis of post-PMII stages provided additional information not obtained by microarrays to understand gene expression and mRNA stability in relation to gene function in the course of pollen-tube extension.

### Comparative analysis of the tobacco male gametophyte and sporophyte transcriptomes

**Figure 3:**
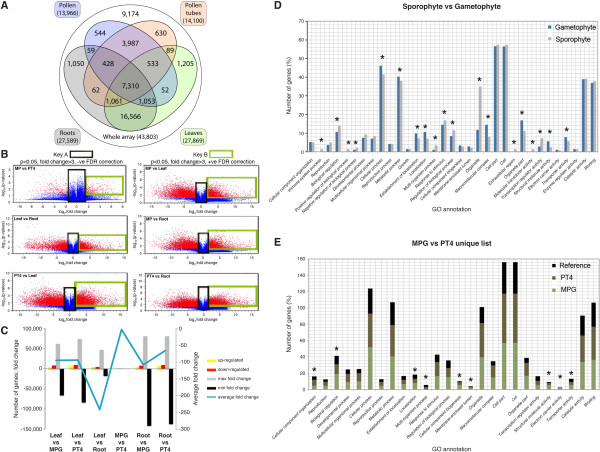
**Differential gene expression and over-represented GO terms between the gametophyte and the sporophyte transcriptomes**. (**a**) Quantification of gametophytic-gametophytic, gametophytic-sporophytic and sporophytic-sporophytic overlaps of transcriptome profiles. Four-way Venn diagram showing all 15 possible overlaps between four independent transcriptome datasets. For each sample, the total number of expressed genes is given in parentheses, together with the total number of unique probes harboured on the array (Whole array). The 9,174 probes were not reliably expressed in any tissue. (**b**) Volcano plot showing pairwise analysis of differentially expressed genes computed through statistical *t*-test of the normalized data. The plot shows differences between the means of the two analysed samples on the x-axis and -log_10 _*p*-values on the y-axis. Probe sets that passed the set criteria (see Materials and Methods section) are highlighted in red (differentially expressed genes), and blue spots represent total probe population. (**c**) Summary of differentially expressed genes following two-way comparison. (**d**) Relative percentage of genes of selected GO categories that were significantly over-represented between the sporophyte and gametophyte transcriptomes. (**e**) Over-represented GO terms of MPG- and PT4-enriched genes not expressed in any sporophytic tissue analyzed. Asterisks indicate GO categories that were significantly different (*p *< 0.05).

Soma-derived tissues constitute a transcriptome that is distinct from that of gametophytes, and thus provide a good reference point to classify genes that are preferentially expressed in the male gametophyte. For systematic comparison of the four tissue samples, only a subset of reliably expressed genes were considered for analysis (see Materials and Methods). On the basis of this criterion, 13,966 probes from MPG (40.3% of expressed genes), 14,100 from PT4 (40.7%), 27,869 from L (80.5%) and 27,589 from R (79.7%) were selected and classified as reliably expressed. A previous analysis of the tobacco transcriptome that utilized 40 k custom-designed Affymetrix microarrays reported reliable expression of 76% of the probe sets in 19 different tobacco tissues, including leaves and roots [19]. We examined the extent of overlap between individual arrays. As anticipated, MPG shared the greatest number of expressed genes with PT4 in comparison with leaves or roots (Figure 3a). The inverse was true between the sporophytic tissues, with > 16,566 (56%) transcripts being exclusive to the sporophyte and shared between leaves and roots (Figure 3a). A detailed examination at the biological significance of these co-expressed genes is discussed below.

When the average of the eight arrays were combined into sporophyte and gametophyte 'master lists' of expressed genes, the reduced complexity of the gametophyte was apparent, with 5,161 probes (14.9% of expressed transcripts) putatively male gametophyte-specific and active in at least one developmental stage. This was significantly less than the analogous value for genes exclusive to sporophytes (18,821 probes, 54.4% of expressed transcripts). The contribution of both MPG and PT4 to the male gametophyte-specific subset was similar, with only 544 and 630 probes exclusive to MPG and PT4, respectively (Figure 3a and Additional file 3: Table S2). This marginal, but significant, change in the pollen-tube transcriptome signified a subtle difference with respect to the diversity of the two transcriptomes and a less significant induction of newly transcribed genes on initiation of the progamic phase. Our results coincide with changes in gene expression observed in Arabidopsis pollen and pollen-tube transcriptomes [9,13], which emphasized that regulation of overall gene expression is similar in species with bicellular and tricellular pollen.

Gene enrichment analysis provides valuable information for prediction of biological functions of gene subsets and dominant pathways imposed in the tissue of interest. We applied a statistical *t*-test on the quantile-normalized log_2_-transformed array data and selected only genes that fulfilled the following criteria: for a gene to have undergone a significant change in expression, a probe must have (1) a *p*-value ≤ 0.05; (2) passed a false discovery rate (FDR) correction to minimize the FDR [20]; (3) an absolute expression signal (fold change) of ≥ 3 (up- or down-regulated); and (4) expression levels of > 100 (arbitrary cut-off). A moderate number of genes (830; 5.3% of male-gametophytic genes) had undergone a significant change in expression between MPG and PT4 (Figure 3b, c and Additional file 4: Table S3). Interestingly, the MPG transcriptome was 11.2% and 10.9% less diverse than the PT4 transcriptome with respect to changes in gene expression when compared with leaves and roots, respectively (Figure 3b, c). For other pairwise analyses, see Additional files 5: Table S4, Additional files 6: Table S5, Additional files 7: Table S6, Additional files 8: Table S7, Additional files 9: Table S8.

We determined whether any GO terms were significantly over-represented among the gametophytic preferentially expressed genes compared with those of the sporophytes. The over-represented GO terms in the gametophyte included those related to localization (GO biological process, *p *= 2.3e-11), transporter and structural molecule activities (GO molecular function, *p *= 1.2e-06 and *p *= 4.4e-16, respectively), as well as membrane-enclosed lumen (GO cellular component, *p *= 9.4e-06) (Figure 3d). The GO categories related to transporter activities (GO molecular function, *p *= 0.0016), calmodulin binding (GO molecular function, *p *= 0.019) and the macromolecular complex (GO cellular component, *p *= 5.4e-4) were among the most over-represented in mature pollen compared with pollen tubes (Figure 3e). Over-representation of these categories in mature pollen, particularly calmodulin protein binding [21,22], may facilitate pollen competence in preparation for initial phases of pollen-tube germination.

### Pollen-tube transcriptome shares a distinct set of genes with roots and root hairs

The characteristic tip growth of a pollen tube shows a common physiology with other polar-growing tissues such as root hairs, trichomes and neuronal axons in animals [23]. We investigated the extent of overlap with the list of genes from Arabidopsis that were either functionally verified to be critical for root-hair development or conferred putative root-hair-specific *cis*-elements (RHE) within their promoters [11,15,16], and references therein]. These motifs were defined by combination of *in silico *(Patmatch Analysis Tool, http://www.arabidopsis.org/cgi-bin/patmatch/nph-patmatch.pl;) and experimental screens that produced an initial list of 904 genes with the consensus RHE element [15]. Although this list was derived from a root-hair-specific transcriptome, the role of the identified genes was not necessarily confined to root-hair morphogenesis. We extended our analysis with the compilation of Cvrčková et al. [16], who gathered a list of genes that had either a root-hair-defective phenotype, root-hair-specific expression and/or were known to have a role in pollen-tube tip growth. This accounted for 73 genes in total [16].

First, we identified genes that overlapped between PT4 and the root transcriptomes of the present study and compiled a set of 9,812 genes (denoted PT4R, Additional file 10: Table S9). To obtain a meaningful and direct evaluation of common pathways involved in tip growth, the PT4R list was filtered further to leave genes with closest Arabidopsis homologs only (e = < 10^-10 ^[19]) (3,264 genes). We designated this subset as PT4R_ATH (Additional file 11: Table S10).

The PT4R_ATH subset was used to screen for overlap with the root-hair transcriptome and proteome [15,16]. We detected 78 genes (8.6%) from the 904 RHE-patched genes that overlapped with the tobacco PT4R_ATH transcriptome, and an additional 26 genes (35.6%) from the list of Cvrčková et al. Collectively, this represented 104 genes (Additional file 12: Table S11). Additional genes with a known function in tip growth were manually selected either without a significant Arabidopsis homolog and/or their expression was confined to 4 h pollen tubes (not reliable in root and root-hair transcriptomes) (Additional file 12: Table S11). Our approach led to identification of genes with a known function in pollen-tube and/or root-hair-cell expansion, as well as novel targets that potentially could play a significant role in polarized cell growth. Several specific targets emerged including: a WD-40 repeat family protein; a RAC GTPase activating protein; a microtubule motor protein; a CBS domain-containing protein; translation initiation factor 5A (eIF-5A); replication factor C1 (AtRFC1); members of the VILLIN and FIBRIN protein families; and copper/zinc superoxide dismutase 1 (CSD1).

To emphasize molecular networks that facilitate polar cell expansion, we investigated GO categories over- or under-represented in the subset of 104 pollen-tube/root-hair overlapping genes. We identified 18 over-represented GO terms, including those associated with transport, cellular component organization, response to stimulus, and nutrient reservoir activity, whereas the GO term 'binding' (ion binding) was under-represented (Additional file 13: Table S12). Highly over-represented subcategories included those associated with chromatin modification (*p *= 0.01, n = 4), translation (*p *= 0.03, n = 4), protein transport and turnover (*p *= 0.013, n = 3), *cis*- and *trans*-Golgi networks, retrograde vesicle-mediated transport, and genes associated with GTPase activities (*p *= 0.003, n = 3), signal transduction (*p *= 0.004, n = 9), microtubule and cytoskeleton organization (*p *= 0.014, n = 3), and cell fate and cell morphogenesis (*p *= 4.15e-05, n = 5). Several of the identified GO terms have been implicated in pollen-tube and root-hair polarized growth [9,13]. In particular, genes involved in chromatin remodeling and transcription, signal transduction and transport previously were reported to be over-represented in pollen tubes that had grown semi-in vivo through the pistil and could thus mediate pollen-tube growth and guidance through the style [9].

### Functional verification of pollen-tube and root-hair overlapping candidate genes using a reverse genetic approach

Five candidate proteins were selected for functional analysis on the basis of their expression profile, GO category, relevance and novelty. Three candidates were identified among GO categories over-represented in the overlap between the pollen-tube and root-hair transcriptomes: Cu/Zn superoxide dismutase 1 (CSD1; GO reactive oxygen species metabolic processes); replication factor C1 (NtRFC1; GO binding); and WD-40 containing protein PD40 (GO intracellular processes, subcategory CUL4-RING ubiquitin ligase complex). The fourth candidate, NTP303 (SKU5-like putative copper oxidase; GO oxidoreductase activities [24]), was selected as the most abundant tobacco pollen-tube wall glycoprotein [25] that is massively translated throughout pollen-tube growth [26] and with predicted important function(s). Finally, eukaryotic translation initiation factor eIF5A (GO primary metabolic processes) was selected as a constitutively expressed protein and a component of the translation machinery during pollen-tube growth.

We used the transfection reagent Cytofectin [27,28] to feed growing pollen tubes with antisense oligonucleotides designed against a target gene. Transfection techniques and microinjection for the introduction of antisense oligonucleotides as well as siRNAs mimic the transfection of *C*. *elegans *and mammalian cell lines with RNA interference (RNAi) sequences [29,30]. The techniques have been used to demonstrate gene function in tobacco and lily pollen tubes [31,32].

#### Transfection with anti-eIF5A and anti-NTP303 does not impair pollen-tube growth

**Figure 4:**
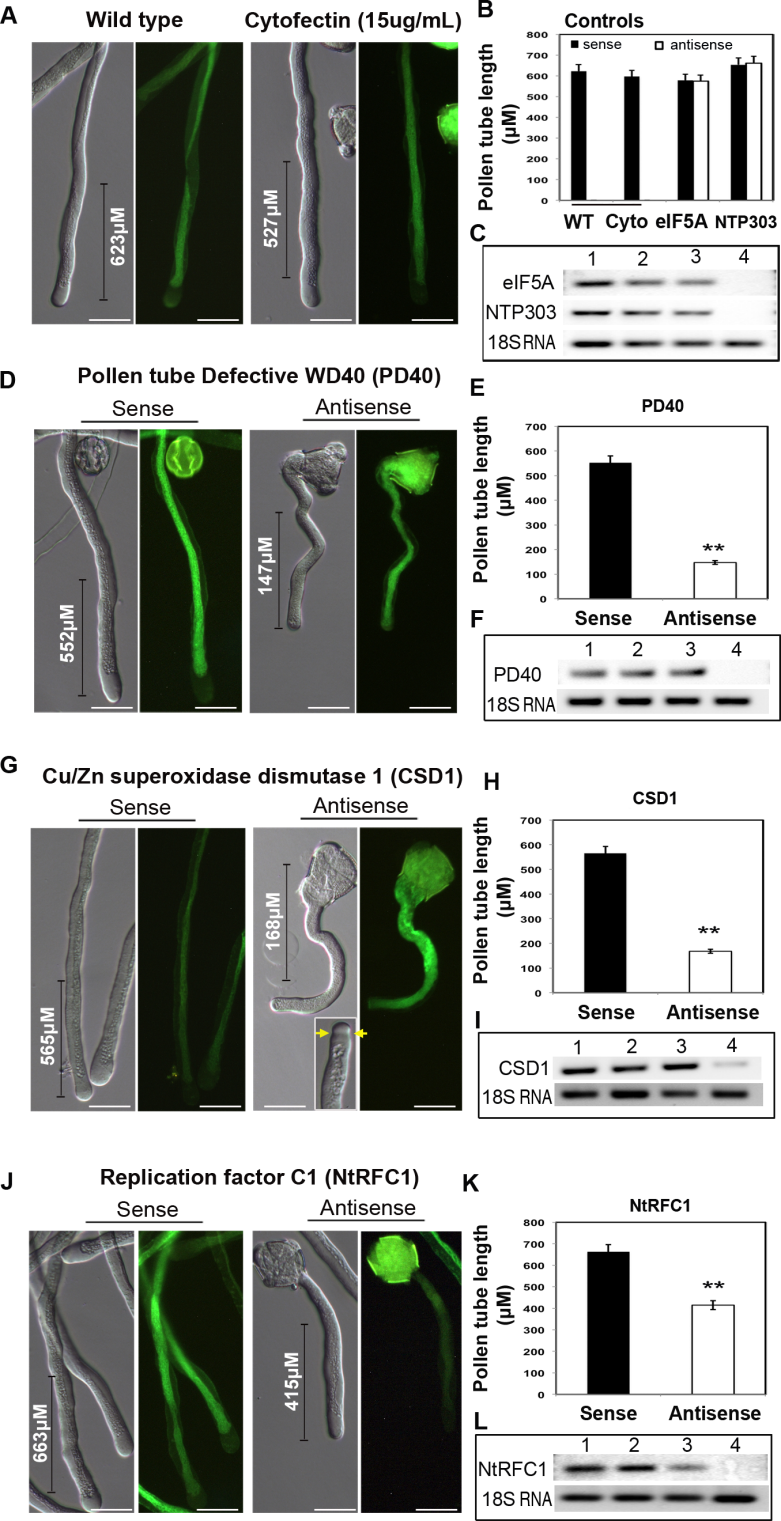
**Antisense knockdown of candidate genes with putative role in polarized pollen-tube tip expansion**. All samples were observed with differential interference contrast (DIC, left panel) and fluorescence microscopy (right panel). The NtpSF3-Lim2a:GFP construct, an actin-cytoskeleton marker [36], was used to monitor possible defects in cytoskeleton organization. (a) Wild-type control and cytofectin-treated pollen tubes. (b) Quantitative analysis of mean pollen-tube length (± SEM) from NTP303 and eIF5A control. Lower panel; semi-quantitative RT-PCR analysis was used to determine transcript levels in each treatment. 1: wild-type control 2: cytofectin-treated pollen tubes; 3: sense-transfected pollen tubes; 4: antisense-transfected pollen tubes. The same numbers key is used for panels (d), (f) and (h). (c, d) Aberrant pollen-tube growth following PD40 knockdown with significant reduction in pollen-tube length (*p *< 0.001), however with no evident defect in cytoskeleton biogenesis or organization. (e, f) Severe pollen-tube growth defects and occasional abnormal tip morphology (inset, yellow arrows) following CSD1 knockdown. Pollen tubes were significantly shorter (*p *< 0.001), with 'hook-like' morphology generated immediately after the initiation of germination. (g, h) Less severe but significant (*p *< 0.01) reduction in NtRFC1-depleted pollen-tube growth. Moderate reduction in transcripts levels were also observed in sense-treated pollen tubes, although this was not reflected by alterations in the pollen-tube phenotype. Scale bar = 20 μM.

Eukaryotic translation initiation and elongation factors have been implemented in various cellular processes, including cell division, cell growth and cell death [33], as well as in plant response to iron deficiency [34]; eIF5A served as a general, non-specific control. No significant difference in pollen-tube length or morphology was observed between wild-type sense- or antisense-transfected pollen tubes after 4 h of pollen-tube cultivation (n = 254, two-tailed Wilcoxon Mann-Whitney *U *test [*U *test hereafter], *p *> 0.05, U = 2932, Figure 4b). With reference to pollen germination dependency on translation [35], we propose a likely redundancy with other translation initiation factors.

The rapid extension of the growing pollen tube is predetermined by the biosynthesis and delivery of pollen tube wall material. NTP303 encodes a 69-kDa glycoprotein [37] assigned a putative function as copper ion oxidoreductase (UniProtKB/TrEMBL, accession B6U720) with predicted localization in the vacuole and chloroplast (WoLFPSORT score = 5 and 4), with a probability of being extracellular (SubLoc RI = 1, Accuracy = 56%). Our attempt to deplete NTP303 gene expression with antisense oligonucleotide transfection resulted in no apparent perturbation of pollen-tube growth or any pleiotropic morphological defects (n = 151, *U *test, *p *> 0.05, U = 309) (Figure 4b).

#### Anti-PD40 induced knockdown displayed severe pollen-tube growth arrest

We investigated the putative role of BP130384 (Arabidopsis homolog AT5G67320), here annotated as PD40 (**P**ollen tube **D**efective WD-**40**), a WD-40 containing protein with predicted nuclear localization (WoLFPSORT score = 8; SubLoc RI = 1, Accuracy = 56%). WD-40 domain-containing proteins are known to be involved in protein-protein interactions that facilitate key developmental processes [38]. Transfection of germinating pollen grains with PD40-antisense oligonucleotides severely perturbed pollen-tube growth (Figure 4c). On average, 36% of PD40 antisense-treated pollen tubes were significantly shorter (n = 252, *U *test, p < 0.001, U = 4694.5) than the average length of the wild-type or sense-treated pollen tubes (Figure 4c, d). Apart from stunted growth, no other morphological abnormalities were observed. Recently, the role of WD-40 proteins as substrate receptors for CUL4-RING E3 ubiquitin ligases has been demonstrated in Arabidopsis and rice [39]. To date, a stand-alone model that involves WD-40 proteins is that of root-hair and trichome specification composed of a WD-40 protein TRANSPARENT TESTA GLABRA1 (TTG1), bHLH transcription factor GLABRA3 (GL3) and two MYB proteins, WEREWOLF (WER) and CAPRICE (CPC), that together impose position-dependent patterning of root-hair specification [40-42]. Our observation places an emphasis on the significance of protein turnover and recycling during polarized cell growth.

#### CSD1 deficiency exhibits a complete block of pollen-tube extension as well as pollen-tube tip morphology

To determine whether our overlapping candidates with predicted role in the regulation of reactive oxygen species (ROS) also play a role in tip growth, we investigated CSD1, an apoprotein of Cu-Zn superoxide dismutase with a known function in ROS detoxification [43]. Recently, CSD1 was demonstrated to be regulated by miR398 at the transcription and translation levels in response to copper availability [44]. We established that CSD1 is cytosol-localized (WoLFPSORT score = 14; SubLoc RI = 3, Accuracy = 84%). Antisense knockdown of CSD1 resulted in stunted pollen tubes with an average length of 168 μM, which was significantly shorter than that of control samples (n = 251, *U *test, *p *< 0.001, U = 4511) (Figure 4e). Approximately 33% of the population displayed the aberrant pollen-tube phenotype (Figure 4f). A small proportion of the pollen tubes (3.4%, n = 91) showed abnormal swollen tips that were not observed in CSD1-sense transfected pollen tubes nor in wild-type pollen tubes (Figure 4e inset). The influence of ROS dynamics on pollen-tube tip morphology has been demonstrated repeatedly and has conclusively highlighted the function of ROS at the apical dome of the germinating pollen tube [10,17]. Our discovery that depletion of CSD1 compromises pollen-tube growth further extends our knowledge of polar cell expansion, and it is likely to have a similar role during root-hair specification and morphogenesis.

#### NtRFC1 knockdown induces partial block of pollen-tube germination

The DNA replication factor C1, NtRFC1 (*Nicotiana tabacum *NCBI accession DW003643, Arabidopsis homolog AT5G22010) mediates genomic stability and transcriptional gene silencing in Arabidopsis [45]. NtRFC1 was predicted to localize in the nucleus (WoLFPSORT score = 12; SubLoc RI = 5, Accuracy = 94%). Pollen germination in the presence of NtRFC1-antisense oligonucleotides for 4 h resulted in pollen-tube extension defects in which 51.7% of the total population had an average length of 415 μM, which was significantly on the dwarf side (n = 191, *U *test, *p *< 0.001, U = 1485) (Figure 4g, h). In the most severe scenario, 6.2% (n = 188) of the pollen tubes showed almost complete arrest in germination. Interestingly, the proportion of defective pollen tubes was enhanced at a higher concentration of NtRFC1-antisense oligonucleotides (data not shown).

The remaining candidates are of considerable interest for further functional analysis to understand the molecular basis that governs pollen-tube tip growth, pollen tube-pistil interaction, signaling and guidance.

### Genes with a demonstrated or potential role in cell-cycle repression are dominant in mature pollen and 4 h pollen tubes

**Figure 5:**
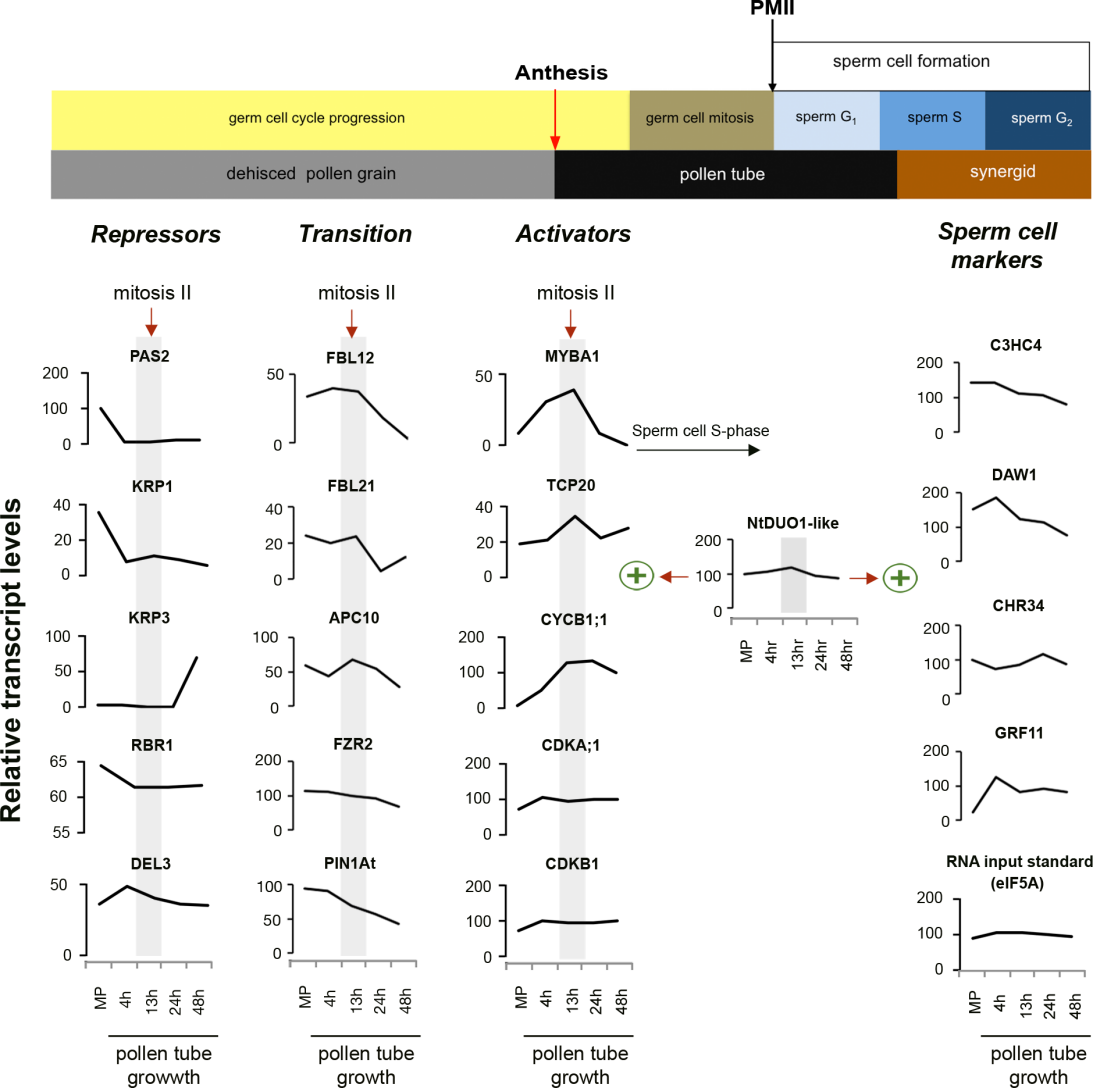
**Expression profiles of core cell-cycle regulators and sperm cell specification markers pre- and post-mitotis of the germ cell**. Schematic representation of tobacco germ cell-cycle progression during pollen-tube growth until near plasmogamy, depicted with reference to DNA content measurements of *Nicotiana tabacum *germ and sperm cells cycle [6]. mRNA expression levels were determined by semi-quantitative RT-PCR with RNA isolated from mature bicellular pollen grains (MPG) and at four stages of pollen-tube growth: 4 h, 13 h, 24 h and 48 h, both pre- and post-PMII. Signal intensities were quantified using Image J software Java 1.6.0 (National Institutes of Health, USA) and normalized against the background signal to obtain relative intensities expressed as relative transcript levels. Genes were classified as *repressors *of G1/S transition; as *transition*, for those that counteract repressors; or as *activators *of G2/M transition and progression. NtDUO1-like is regarded as an integrator of germ cell cycle mitotic progression and sperm cell fate specification, with functional evidence from the Arabidopsis male gametophyte [47]. Sperm cell fate markers are also reliably expressed pre and post germ cell mitosis to near karyogamy. Note: RBR1 levels are never minimal, accentuating the primary role of RBR1 in maintaining the vegetative cell nucleus at G_0 _[48].

We used MAPMAN software http://mapman.gabipd.org/web/guest/mapman[46] to extract a cluster of genes classified as regulators of cell division and cell-cycle progression. We limited our analysis to genes reliably detected in both replicates and with expression levels well above the background. Thirty-seven genes were grouped with a role in cell-cycle progression, whereas 25 genes were annotated to participate in some aspects of cell division (Additional file 14: Table S13). We used semi-quantitative RT-PCR to validate the expression of selected target genes and to extend the analysis post-PMII after 13, 24 and 48 h of pollen-tube growth (Figure 1c, Figure 5). The sample selection allowed us to establish the expression dynamics of these key cell-cycle regulators pre- and post-PMII in angiosperms with bicellular pollen.

#### Reduced accumulation of cell-cycle repressors implies derepression and mitotic entry of the germ cell

Quantification of DNA content in bicellular pollen indicated that the progression of the germ cells requires cell-cycle arrest or slow progression through the S-phase immediately after the first haploid division of the microspore until the entry into PMII, 8-12 h after pollen germination [5,6]. Our analysis revealed a dominant presence of known cell-cycle inhibitors at the mature pollen stage that persisted into 4 h pollen tubes (Figure 5, Additional file 14: Table S13). These inhibitors included a RETINOBLASTOMA protein 1 (RBR1), two inhibitory kinases of cyclin-dependent kinases--namely, Kip-related proteins 1 and 3 (KRP1, KRP3)--as well as DEL3 and PAS2. We previously demonstrated the essential role of RBR1 in blocking S-phase entry of the vegetative-cell nucleus and linked this later role with an earlier function of specifying cell fate of the vegetative cell and the germ cell in the Arabidopsis male gametophyte [49]. Our present results showed that RBR1 transcripts are highly abundant at the mature pollen stage. Its expression is reduced after 4 h pollen-tube growth and remained at steady-state levels up to 48 h pollen tubes (Figure 5). Similarly, KRP6/7 are known inhibitors of germ-cell-cycle progression in Arabidopsis and their repression activities are suppressed by an F-box protein, FBL17 [50]. In the present study we detected two members from the KRP family, KRP1 and KRP3, with opposed expression profiles. Whereas KRP1 showed high abundance in mature pollen that dramatically declined thereafter up to the 48 h time point of pollen-tube growth (Figure 5), KRP3 showed very low expression throughout development before significantly peaking in 24 h pollen tubes (Figure 5).

Transition to cell-cycle progression is achieved by blocking the activities of the cell-cycle repressors. A family of F-box proteins, components of the SKP1-CUL1-F-box protein complex (SCF), is known to counteract the repression of CDKA induced by KRP proteins. We detected the expression of five F-box-like genes (Additional file 14: Table S13). Semi-quantitative RT-PCR analysis showed that the expression of FBL12 and FBL21 peaked at the 'division window' followed by a rapid decline post-PMII (Figure 5). We also detected expression of APC10, a component of the anaphase-promoting complex (APC/C) involved in the turnover of cyclin subunits through ubiquitin-mediated proteolysis of cyclin proteins to prevent premature entry and to promote exit from the mitotic cycle. Recent genetic characterization of another APC/C component, *APC8*, has linked APC activities to the transcriptional repression of CycB1;1 by activation of a repressor of DUO POLLEN 1, miR159 [51]. Our analysis showed that APC10 expression levels were reduced until PT4, and then increased significantly to maximum levels at the 13 h time point (Figure 5). This observation points to the dual role of APC/C in the germ cell cycle, in which CyclinB1 transcriptional repression is coupled with ubiquitin-mediated degradation in the male gametophyte.

#### A significant peak of G2/M factors marks transition to activation and germ-cell mitotic division

The dormant state of the mitotic cell cycle in 4 h pollen tubes was further emphasized by the under-representation of known cell-cycle activators. A G2/M master regulator, CycB1;1, was only unreliably detected in mature pollen and 4 h pollen tubes (Additional file 14: Table S13). Inversely, at the transition to the second mitotic division of the germ cell (~10-13 h after in vitro pollen germination), CycB1;1 expression was specifically and dramatically enhanced at the 'division window' (cca ~10-13 h after in vitro pollen germination) and maintained its status before gradually decreasing thereafter (Figure 5). We also detected two MYB transcription activators of CycB1;1 expression: NtMybA1, and a putative tobacco ortholog of Arabidopsis DUO POLLEN1 (DUO1), here annotated as NtDUO1-like (NCBI blastp accession AB032537 (Q8H0H3) [52], e-value of 1e-78). The tobacco NtmybA1 is a R1R2R3 MYB transcription factor similar to animal c-Myb proteins that induce CycB1;1 expression specifically at the G2/M phase of the cell cycle [53,54]. The role of NtmybA1 in the activation of CycB1;1 is enhanced specifically at G2/M by TCP20, a teosinte-branched cycloidea PCNA factor, through binding on enhancer elements (GCCCR) within CycB1;1 promoter sequences [55]. DUO1, on the other hand, is an R2R3 MYB transcription factor that we previously demonstrated to be a dual regulator of germ-cell division and sperm cell fate differentiation [47,56]. All three factors, NtMYBA1, NtDUO1-like and TCP20, were gradually enhanced, and simultaneously peaked at the 'division window', which mirrored the profile of their target (CycB1;1) and the cell-cycle status of the germ cell (Figure 5). Remarkably, the expression of these mitotic entry gate-keepers reached a maximum level at the 13 h time point, by which time > 80% of the germ cells are in the transition through mitosis or had completed mitotic division to produce the two sperm cells (Figure 1c, inset).

The stable presence of other key positive regulators, such as CDKA;1, CDKB1;1 (which counteracts KRPs activities on CDKs [57]), and CDKB2;2 (a partner of CycD3 that promotes S-phase entry and progression), appears to provide a positive balance and a molecular switch preset to enter and progress through the cell division cycle (Figure 5, Additional file 14: Table S13). We observed a marginal increase in expression of CDKA;1 and CDKB1;1 from mature pollen to PT4 preceding the strong expression of CYCB1;1 (Figure 5). Their expression was stabilized thereafter throughout the progression of the progamic phase.

#### Sperm cell fate specification markers continuously accumulate pre- and post-division of the germ cell

The coupling of the germ-cell mitotic division with sperm cell fate specification is designated by the overlapping expression of known cell fate differentiation markers within the germ cell that progresses to the newly formed sperm cells. We showed previously that DUO1 integrated both events, which enabled germ-cell division and regulation of the transcription of a repertoire of sperm-cell differentiation markers [47,56]. In this study, we detected 29 tobacco homologs of Arabidopsis DUO1-induced targets including two verified targets (Additional file 15: Table S14 [56]). Among these homologs is a chromatin remodelling protein (CHR34), WD-40 CUL4-RING ubiquitin ligase (DAW1), a zinc finger family protein (C3HC4-type RING finger), DUO1 Activated Unknown protein (DAU1), a NAC domain-containing protein 74 (anac074), a sugar transporter (AT5G26250), and a heavy-metal-associated domain-containing protein (AT1G51090). Given the demonstrated role of DUO1, these genes are also likely to be regulators of sperm cell fate specification in tobacco. We verified expression of four candidates and established that C3HC4, CHR34 and GRF11 showed similar abundance and expression profiles, whereas DAW1 peaked at PT4 and dropped to comparable levels thereafter (Figure 5, Additional file 15: Table S14). Additional target genes are almost certainly expressed in the tobacco male gametophyte and might play a more conserved role to that in Arabidopsis, including the determination of sperm cell fate and function.

In summary, our analysis of male gametophyte development pre- and post-second haploid mitosis uncovered an alternating accumulation of conserved regulators of cell proliferation and cell fate determinants in synchrony with the progression of the germ cell cycle. Their activities are the likely cause of the heterochronic shift that has led to diversified patterns of spermatogenesis and gamete fusion among angiosperms.

## Discussion

With continuous improvement in gene annotation and the availability of functional analysis tools, *Nicotiana tabacum *has emerged as a'practical' experimental model for investigation of gene function, particularly of genes involved in pollen-tube growth and fertilization. Thus, our data set offers new opportunities for cross-species functional studies - for example, extended studies of lily GCS1 [58,59] in organisms with advanced genomic information available, such as Arabidopsis and rice. Although markedly improved, incomplete genome annotation in Solanaceae species has remained a drawback for research on these plants.

### Comparative analysis of pollen-tube and root-hair transcriptomes provides insight into the genetic network of cell tip expansion and morphogenesis

We identified a suite of genes from a global-comparative analysis of pollen-tube and root-hair transcriptomes, with the implication of uncovering the genetic network involved in the control of tip-growth morphogenesis. Of the genes identified (~104), 18 GO categories were classified as highly over-represented (*p *< 0.05), which included those associated with chromatin modification, transcription and translation, protein transport and turnover, *cis*- and *trans*-Golgi networks, retrograde vesicle-mediated transport, signal transduction, microtubule and cytoskeleton organization, cell fate and cell morphogenesis, and cell and cell-wall metabolic processes. Several genes emerged from the identified GO categories were functionally characterized previously. These include two transcription factors - NPR1-like protein 3 (TRAF family) and RAP2.12 (AP2-EREBP family) - that are implicated in defence responses and enhanced ADH1 transcription and enzyme activities [60], respectively. Using the pollen tube as a model for functional tests, we verified the function of three candidate genes and illustrated their role in pollen-tube tip growth (Additional file 13: Table S12). Our results demonstrated that copper metabolism (CSD1), chromatin remodelling (AtRFC1), and regulated ubiquitin-mediated proteolysis (PD40) are essential processes during pollen-tube extension. Similarly, recently published analyses of F-box proteins SKIP2 (Arabidopsis SCF-type F-box and leucine rich repeat-containing E3 ubiquitin ligase, VFB-4) and LSK1-LSK3 (closest lily homologs of Arabidopsis ASK1) also demonstrated the importance of 26S proteasome-mediated proteolysis during pollen-tube extension [61]. Furthermore, expression 'late genes' is known to persist at a certain rate during pollen germination [13,62,63]. NtRFC1 has been implemented in maintaining genome stability and transcriptional gene silencing [45], and thus could function to repress the expression of germ-cell genes within the vegetative cell and simultaneously to regulate the tobacco germ-cell chromatin status. The concept of the two cell types sharing resources and regulatory molecules through cytoplasmic bridging potentially to implement synchronized control during maturation is increasingly apparent, although it has yet to be demonstrated at the molecular level [64,65]. In the present study, we demonstrated the essential role of NtRFC1 as a component of the gene expression network in association with pollen-tube tip expansion.

We acknowledge that our approach could have been too stringent on selection criteria and resulted in potential candidate losses. For instance, three genes - JACKDAW (JKD), CTR1 and AXR2 - are essential components of the genetic network that regulates root-hair-cell specification and extension [41,66,48], but their expression in our data set was classified as unreliable, most likely because of their low expression levels. On the other hand, the identification of genes such as GNOM-like 1 (NtGNL1), a key component of pollen-tube tip-defining growth [67] in the tobacco pollen-tube transcriptome, further endorsed our selection criteria. We simultaneously detected Cyclophilin 5 (CYP5), DV999409, which is known to interact with GNOM in vitro [48]. Moreover, our identification was not only limited to cell-fate-specification genes, but also included genes required for the proper expansion of most cell types, such as members of the root-hair defective 3 GTP-binding (RHD3) protein family (Additional file 12: Table S11) [67,68]. Our data offer further extension to investigate the control of tip specification, initiation and expansion, and provide a platform by which microarray-directed reverse genetic analysis can be used to enhance our understanding of polarized cell expansion.

The key question remains how these genes reprogram pollen-grain polarity in response to hydration to mediate the point of progamic phase initiation and pollen-tube growth through its multiple phases within the female transmitting tissues. This comparative approach supports the idea that, by investigation of the genetic overlap between polarized pollen tubes and roots/root hairs, it is more than likely to uncover universal mechanisms employed to promote tip expansion in order to achieve the intended morphological structure and cell function.

### Lack of phenotypic defects following *NTP303 *knockdown suggests existence of robust backup for key regulators of pollen-tube growth

The NTP303 glycoprotein is the most abundant protein in 4 h pollen tubes [37] and is a major component of the pollen tube wall [25]. Previous attempts to knockdown *NTP303 *expression *in planta *using an RNAi vector did not alter pollen development or pollen-tube growth. However, targeting NTP303 family members (*ntp101*, *201*, *302 *and *ntp805*) simultaneously produced male-sterile plants that showed pollen-tube growth defects in vivo but not in vitro [69]. This observation led to the hypothesis that NTP303 could (1) facilitate pollen-tube growth through the style, and hence the effect could only be observed in vivo, although NTP303 protein was not detected in transmitting tissues; and/or (2) participate in pollen-tube wall formation, and thus its knockdown could only result in weak cell-wall formation that is not evident in vitro. *In planta *and in vitro studies using amino acid microsequencing and immunolocalization approaches reported NTP303 protein detection (1) on vegetative plasma membranes surrounding the vegetative cell, generative cell and sperm cells of pollen and pollen tubes; (2) in the pollen-tube cell wall and callose plugs; and (3) in pollen-tube pellet fractions after 1 h of pollen tube growth and in cytosol after 8 h of pollen tube growth [69]. These results strongly suggested that NTP303 is translocated to the pollen-tube cell wall/membranes possibly by vesicular trafficking [69]. However, NTP303 was not detected at the tip of the growing pollen tubes, and thus it is unlikely to be involved in direct signal transduction. On the other hand, it could be part of the intracellular signalling system, because both the GTP-binding site and several possible phosphorylation sites are predicted in addition to transit peptide [70]. NTP303 is homologous to Arabidopsis SKU5 (SKS12, AT1G55570), a member of the small SKS family (SKS11, 12, 13 and 14) that possesses an ascorbate oxidase motif and encodes an extracellular glycosyl phosphatidylinositol-anchored glycoprotein [71]. This motif is required for tobacco BY2 cell expansion, and a *sku5 *mutant displayed growth defects in directional root growth [71]. Our observation of the lack of pollen-tube defects after transfection with NTP303-antisense oligoneucleotides repeated and independently verified previous observations [69]. Given that NTP303 is the most abundant component of the pollen tube, NTP303 mRNA distribution into storage compartments [62] (Hafidh, Čapková and Honys, unpublished data) and its regulation at the transcription and translation level [72,73], together with its expression and functional overlap with other family members, might provide a robust 'back-up' to bypass an effect from a 'defective member' and ensure pollen germination and successful fertilization. Our current attempt to manipulate NTP303 at the protein level appears to be more effective, and thus might be more informative for elucidation of the precise function of NTP303 (Čapková, personal communication).

### Determining the conserved pathway of germ cell division and sperm cell differentiation in angiosperms; a genetic model from a cell cycle perspective

**Figure 6:**
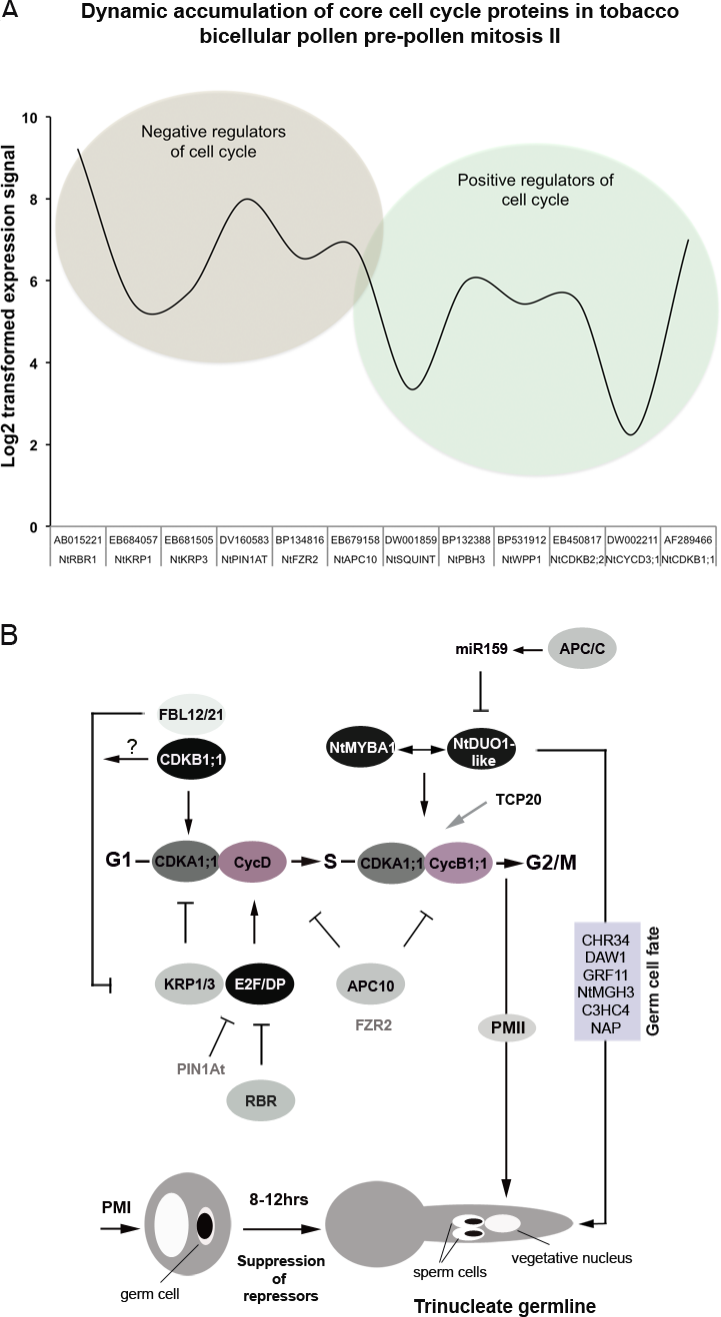
**Putative core cell-cycle model Imposed the heterochronic shift during angiosperm spermatogenesis and fertilization**. (**a**) Representative dynamic expression of cell-cycle genes at the time of anthesis of tobacco bicellular pollen as derived from transcriptomic data. The relative abundances between negative and positive regulators gives a snapshot of the cell-cycle status of germ cell and vegetative cell. Increased expression of positive regulators and post-translational inhibition of the repressors signifies the progression of the germ cell cycle to enter PMII for sperm cells production. This progression is in parallel with the expression of germ-cell fate determinants integrated by the NtDUO1-like Myb transcription factor and in synchrony with pollen-tube growth rate through the female pistil, with the effect of synchronizing the gametes cell-cycle progression for successful karyogamy. (**b**) Depicted model of core cell-cycle regulatory network likely to account for the heterochronic shift in spermatogenesis among flowering plants. The model was derived on the basis of the combined expression analysis presented herein. Key other proteins and family members are also expected to be involved in this network. Note: the hierarchical relationship between R2R3 NtDUO1-like and NtMYBA1 (both activators of CycB1;1 transcription) is yet to be established, together with that of three other R1R2R3 plant Myb transcription factors.

Limited studies of sperm-cell DNA content in developing pollen and in the pollen tube from inception through entry into the embryo sac have been frequently used as the basis for predictions of the expression of cell-cycle-related genes, specifically CDKs and cyclins [5,52,74]. Transition through cell cycle phases in plants is primarily controlled, as in yeast and mammals, by the interactions of CDKs with the regulatory cyclins subunit. CDKA genes are constitutively expressed throughout the cell cycle, and they interact with several cyclin proteins to regulate transition through phases of the cell cycle, including the germ cell cycle in Arabidopsis [75]. Plants bearing the *cdka;1 *mutation are occasionally defective in germ-cell division, and thus fail to produce the two sperm cells necessary for the double fertilization [74,76-78]. We recently showed that the *cdka;1 *single sperm cell is not impaired in the expression of sperm cell fate markers, which is indicative of the competence of the sperm cell produced [47]. Interestingly, the single sperm cell produced is able to fertilize either of the female gametes and produces equal proportions of seeds with either a single endosperm or single embryo [74]. Despite the occurrence of double fertilization in the presence of *cdka;1 *sperm cells, the central cells do not undergo karyogamy, which results in impaired endosperm development and eventual seed abortion [74]. This lack of nuclear fusion might potentially be because of lack of cell-cycle synchrony between the central cells and the *cdka;1 *sperm cell at the time of karyogamy. Cell-cycle checkpoints (together with the rate of cell-cycle progression) are maintained by a repertoire of repressor proteins, including Retinoblastoma-related protein (RBR), Kip-related proteins (KRPs), and inhibitors of CDKs activities (ICKs). RBR1 plays an essential role in the specification of vegetative cell and germ cells and in the control of cell proliferation and differentiation [49], and has an extended function in the regulation of Polycomb Repressive Complex 2 (PRC2) and METHYLTRANSFERASE 1 (MET1) in male and female gametophytes [70]. Activity of these proteins is post-translationally modulated by either phosphorylation or protein turnover, by the ubiquitin-mediated proteolysis complexes, SCF and APC. Interestingly, *apc8 *mutation inhibits germ cell division, whereas *apc8*/*apc10 *hypermorphic lines showed endoreduplication in rosette leaves of *Arabidopsis thaliana *and pleiotropic morphological defects, including small siliques, curled leaves and abnormal phylotaxy [79]. Our GO annotation of cell cycle genes (Additional file 14: Table S13) and semi-quantitative RT-PCR analyses (Figure 5) combined with previous knowledge on germ cell-cycle control [80] enabled us to propose a hypothetical genetic model from a tobacco male germ cell-cycle perspective. The proposed model is reminiscent of a biological 'clock' by which male and female gametes synchronize cell-cycle progression and coordinate gametogenesis events for successful fertilization (Figure 6). It not only provides a foundation to understand the molecular basis of the evolution of pollen tricellularity, but also acts as an informative 'genetic map' for experimental models to produce apomorphic tricellular pollen and to inspire current in vitro fertilization protocols to produce hybrid plant species. Thus understanding of this regulon could provide a useful tool for plant breeders and conservationists to utilize, either to avoid hybridization or to promote crossing of distantly related plant species that are otherwise separated because of hybrid infertility.

## Conclusions

In this study we generated and verified transcriptomic profiles for *Nicotiana tabacum *mature pollen and growing pollen tubes. We functionally demonstrated the role of three pollen-tube-expressed genes in the flawless progression of the progamic phase. We further defined a gene subset, the expression of which overlaps between two tissues characterized by polarised tip growth--namely, pollen tubes and root hairs. We provided additional evidence that knockdown of the tobacco pollen-specific protein NTP303 does not cause apparent phenotypic defects and that its function is sufficiently backed up by its related family members. In addition, we demonstrated expression profiles of known cell-cycle regulators in the tobacco male gametophyte and proposed a genetic model that underlines the regulation of cell-cycle progression during haploid pollen mitosis II as a basis of pollen bicellularity in *Nicotiana tabacum*.

By making the tobacco pollen transcriptomic data publicly accessible, we offer a solid reference benchmark for future transcriptome-based functional studies. The generated transcriptomic data sets have the potential, together with other already available gene expression profiling resources, to provide the foundation for future studies of the evolution of pollen tricellularity, which has occurred independently in several angiosperm lineages. The data sets also inspire development and improvement of current in vitro fertilization protocols that are of great interest not only to plant researchers but also to breeders and conservationists. Successful protocols will offer an opportunity to cross and generate hybrid lines that otherwise face cross-compatibility barriers.

## Methods

### Plant materials and growth conditions

Wild-type tobacco plants (*Nicotiana tabacum *cv. Samsun) were used for the collection of tissue samples for all downstream studies. Seeds were sown in a greenhouse under short-day conditions at 22-25°C. Adult plants with a fully developed root system were transplanted to an outdoors greenhouse on ground compost and grown under the natural day-night photoperiod in spring and summer. Pollen grains were collected throughout the season and their germination rate was monitored (Figure 1)

### Collection of pollen and in vitro pollen-tube cultivation

Mature pollen was isolated aseptically as described previously [81]. Flowers were collected 1 day before anthesis. Stamens were removed from the flowers into a Petri dish to dehisce in a fume-hood overnight at room temperature. Dry pollen grains were then filtered through a nylon mesh (Miracloth, pore size 50 μm), weighed and stored at-20°C. Over 80% of the collected pollen grains could successfully germinated and give rise to a pollen tube, irrespective of the collection date (Figure 1).

For in vitro pollen tube germination, approximately 10 mg/10 mL pollen was resuspended in pollen germination media (SMM: 0.3 M sucrose, 1.6 mM H_3_BO_3_, 3 mM Ca(NO_3_)_2 _4H_2_O, 0.8 mM MgSO_4_.7H_2_O, 1 mM KNO_3_) and aliquoted into conical flasks. The 24 h and 48 h pollen tubes were cultivated with SMM medium supplemented with casein (1 mg/mL). Cultures were incubated in a water-bath shaker at 140 rpm for 2 h and then slowed down to 90 rpm for the remaining cultivation time at 26°C in the dark. Similar procedures were followed for other pollen-tube cultures (13 h, 24 h and 48 h cultivation), though under sterile conditions. Aliquots of the samples were stained with aniline blue and 4',6-diamidino-2 phenylindole (DAPI) staining, and analyzed under a light microscope (Figure 1). Pollen tubes were vacuum-filtered, flash frozen in liquid nitrogen and stored at -80°C prior to RNA extraction. Sporophytic tissues (leaf discs and roots) were collected from juvenile plants and also from excavated adult plants. Collected samples were immediately frozen in liquid nitrogen.

### RNA extraction, probe preparation and microarray hybridization

Total RNA was isolated using the Qiagen RNeasy Plant Kit in accordance with the manufacturer's instructions (Qiagen, Valencia, CA) and treated with DNaseI (Promega. Madison, WI). RNA was quantified using NanoDrop (Thermo Scientific, Wilmington, USA). Prior to shipment, five replicates from each sample were tested using semi-quantitative RT-PCR with two marker genes, Nt-eIF5A and a constitutive 18S rRNA, for reproducibility. RNA concentration, purity and integrity (RIN) were assessed using an Agilent 2100 Bioanalyzer (Agilent Technologies, Boblingen, Germany) at ImaGenes (Berlin, Germany). Biotinylated target cRNA was prepared from 50 ng of reverse-transcribed total RNA (One-Cycle Target labelling and control reagents; Agilent Technologies). Labeled cRNA was fragmented and 15 μg were used for Agilent 44 K tobacco microarray hybridization. Hybridized chips were scanned on an Agilent High Resolution Microarray Scanner.

### Bioinformatics and statistical analysis of Agilent 44 K tobacco genome array data

All transcriptomic data sets were normalized using freely available dChip 1.3 software http://www.dchip.org. The reliability and reproducibility of the analyses were ensured by the use of duplicates in each experiment, the normalization of all arrays to the median probe intensity level, and the use of normalized intensities of all arrays for the calculation of model-based gene-expression values based on the Perfect Match-only model [82]. For each sample, only probes with the detection call of 'present' and an expression value detection level of 'well above background' (Boolean flag, two-sided *t*-test) in both replicates were considered to be expressed.

To determine the quality of the quantile-normalized data set and the correlation between arrays, CLC Genomics Workbench version 4.5.1 (CLC bio, Aarhus, Denmark) was used to compute mean expression values and corresponding *p*-values of the log_2_-transformed data. The output of this analysis was used to assess the correlation between samples using PCA, as well as independently using hierarchical clustering. To observe the variance of the distribution of the mean expression levels, scatterplots were used for pairwise comparison between samples.

Genes differentially expressed between arrays were statistically determined using the following criteria: for all filtered quantile-normalized datasets, (1) probes must have *p*-values of < 0.05; (2) the *p*-values must pass FDR correction to minimize false discovery rate; (3) they should have an absolute expression value (fold change) of ± > 3; (4) and they must have expression levels of > 100, well above background. All genes that passed the above test from pairwise comparisons are reported in Additional file 4: Table S3, Additional file 5: Table S4, Additional file 6: Table S5, Additional file 7: Table S6, Additional file 8: Table S7, Additional file 9: Table S8, together with the results from the statistical analysis.

### Gene ontology analysis for PT4R overlapping genes and the gametophyte-specific subset

Gene ontology term enrichment analysis was performed using the g:Profiler http://biit.cs.ut.ee/gprofiler/ and GoToolBox http://gin.univ-mrs.fr/GOToolBox, gene ontology databases, essentially as described in [83,84], together with application of the hypergeometric distribution test, and Benjamini and Honchberg corrected *p*-value cut off < 0.05 [20]. GO terms from the categories 'biological processes', 'molecular function' and 'cellular activities' with an adjusted *p*-value of < 0.05 were considered over-represented in a subset of genes analyzed. For gametophyte and sporophyte preferentially expressed genes, enriched GO terms are shown in Figures 3d and 3e. PT4R over-represented GO categories are provided in Additional file 12: Table S11.

### Real-time quantitative RT-PCR and semi-quantitative RT-PCR analysis

Independent verification of the expression profiles predicted by microarray was conducted using the same pool of RNA with qRT-PCR and semi-RT-PCR. For each replicate, 500 ng of total RNA was treated with DNAseI (Promega), and 50 ng was reverse-transcribed with ImProm-II reverse transcriptase (Promega). Real-time RT-PCR was performed using the LightCycler 480 SYBR Green I Master Kit (Roche Diagnostics, Mannheim, Germany) in a 480 LightCycler system (Roche Diagnostics). The gene encoding 18S rRNA was used to normalize the expression of all genes examined. For semi-RT-PCR of the cell-cycle and cell-fate genes, 1/10 dilution of the cDNA generated was used as a template for a 30-cycle PCR reaction. The thermocycler program was as follows: 2 min at 95°C; 45 cycles of 30 s at 95°C; 30 s at 55°C; 30 s at 72°C; and 5 min at 72°C. eIF5a was used as a standard control for RNA input. All primers used in these experiments were designed using primer 3 software http://frodo.wi.mit.edu/primer3/, and the sequences are available for download as Additional file 16: Table S15.

### Antisense-mediated knockdown of selected target genes

NCBI-derived sequences of all selected tobacco target genes were used to design corresponding antisense and sense oligodeoxynucleotides for functional analysis. Antisense oligonucleotides can interfere with gene function through RNase H-mediated mRNA degradation, translation repression or alteration of splicing [85]. cDNA sequences of each target gene were submitted to Integrated DNA Technology (IDT, http://eu.idtdna.com/home/home.aspx) and Soligo S-fold software http://sfold.wadsworth.org/cgi-bin/index.pl to compute optimal antisense oligonucleotides and predict the most accessible target sites based on Matveeva rule set and Soligo algorithms, respectively. Two pairs were designed for each gene. 21-mer oligonucleotides corresponding to antisense and sense (control) were synthesized with 5'-3' phosphorothiote modification (three bases from each end, Generi-Biotech, Hradec Králové, Czech Republic). All oligonucleotides were OPC-purified (Oligonucleotide purification cartridge). The sequences of the antisense oligonucleotides used were as follows: PD40 (NCBI; BP130384), sense: 5'-ACTCCACACACACAGAGCCT-3', antisense: 5'-AGGCTCTGTGTGTGTGGAGT-3', NTP303 (NCBI; X69440), sense: 5'-CACCCAATGAAATTAGTCGA-3', antisense: 5'-TCGACTAATTTCATTGGGTG-3', ATRFC1 (NCBI; DW003643), sense: 5'-ACGAGTTACGATCAGTCGAC-3', antisense: 5'-GTCGACTGATCGTAACTCGT-3', eIF5a (NCBI; CV021663), sense: 5'-ACTACATTCGAAGCTCTAGC-3', antisense: 5'-GCTAGAGCTTCGAATGTAGT-3', Cu/Zn superoxide dismutase 1, CSD1 (NCBI; EB439640), sense: 5'-AACGGGACCACATTATAATC-3', antisense: 5'-GATTATAATGTGGTCCCGTT-3'.

### Pollen-tube deoxyoligonucleotide transfection

For each pair of sense and antisense oligonucleotides, 15, 30 and 50 μM were mixed with Cytofectin (Genlantis, San Diego, CA) according to Moutinho et al. [27], to a final volume of 100 μL. A concentration of 30 μM was determined to be sufficient for most genes, with the exception of ATRFC1. Pollen tubes were transfected and germinated on a microtitre plate under constant shaking (140 rpm) at 27°C in the dark. Initial screening for phenotype was done in the microtitre plate; thereafter, an aliquot from each sample was taken for further microscopic analysis. For each gene tested, including controls, pollen tubes were cultivated with and without the cytofectin/deoxyoligonucleotide mixture for 4 h in vitro and then fixed with 4% formaldehyde prior to length measurements. Two biological and three technical replicates were performed for each experiment, and mean values are presented.

### Microscopy

For nuclear visualization, DAPI staining solution (0.1 M sodium phosphate, pH 7; 1 mM EDTA, 0.1% (v/v) Triton X-100, 0.8 mg/ml DAPI) was used to stain dry mature pollen grains and germinated pollen tubes [86]. For the knockdown experiments, pollen tubes were first fixed with 4% paraformaldehyde, 50 mM PIPES and 10% sucrose, vacuum infiltrated for 5 min and incubated for 1 h. Pollen tubes were then washed three times with 50 mM PIPES (pH 6.9). Light and fluorescence microscopy was carried out with a Nikon TE2000-E fluorescent microscope (Nikon, Japan) using DAPI and GFP filter sets. Image capturing and processing was carried out and pollen-tube length measurements were taken using NIS-Elements AR3.0 software (Nikon instruments, Melville, USA) and Adobe Photoshop http://www.adobe.com.

## Abbreviations

MPG: dehisced mature pollen grain; PT: pollen tube; oligo: soligonucleotides; FDR: false discovery rate; GO term gene ontology.

## Additional files

The following additional data are available with the online version of this paper. Additional data file 1 is a table of complete data set generated in this study with mean normalized expression derived from two biological replicates and with expression levels 'well above background'. Additional file 2: Table S1 is a table gametophyte specific expressed genes. Additional files 3: Table S2, Additional files 4: Table S3, Additional files 5: Table S4, Additional files 6: Table S5, Additional files 7: Table S6, Additional files 8: Table S7 are tables of differentially expressed genes between [1] MPGvsPT4 [2] leaves vs MPG [3] roots/root-hairs vs MPG [4] leaves vs PT4 [5] roots/root-hairs vs PT4 and [6] leaves vs roots/root-hairs, respectively. Additional file 9: Table S8 is a list of PT4-root/root-hairs overlapping subset of genes (PT4R). Additional file 10 Table S9 is a similar list as that of Additional file 9: Table S8 with identified Arabidopsis closest homologs (PT4R_ATH). Additional file 11 is a list of PT4R_ATH genes that were identified to overlap with Arabidopsis root-hair trichoblast and virtual proteome. Additional file 12: Table S11 is a list of overrepresented GO terms for genes that showed overlapping expression between PT4R_ATH and Arabidopsis root-hair trichoblast. Additional file 13: Table S12 is a list of identified cell-cycle genes expressed in the MPG and PT4 pollen stages. Additional file 14: Table S13 is a table of DUO1 target genes that were detected in our pollen and pollen tube transcriptomes. Additional file 15: Table S14 is a list of all primers used in this study.

## Authors' contributions

SH and DH designed the study. SH, KB, PR, JF and VC performed the experiments. SH and DH analysed the data. SH wrote the manuscript, which was further edited by SH, VC, KB and DH. All authors read and approved the final manuscript.

## Supplementary Material

Additional file 1 **Figure S1 Data normalization and analysis of replica reproducibility**. (**a**) Box plot of the raw hybridization signal from the four samples, highlighting systematic differences between samples. The inset shows the same data after the normalization. (**b**) Output of the first and second principal component (PCA) analysis of the log_2_-transformed data sets. The largest and second-largest principal component (variability projection 1 and 2, respectively) are displayed in orthogonal directions, assessing the overall homogeneity between replicates and variability between samples of different tissue types as reflected in their grouping. The inset shows hierarchical clustering of the same data set grouped according to array similarities and differences. Since both methods produced identical grouping patterns, the quality of the dataset was considered reliable and useful for comparative analysis. (**c**) Scatterplots of log_2_-transformed microarray data showing a correlation between tissue arrays. A wider symmetrical scattering of the spots from the 'trendline' implicates higher variability between the tissues, with less dependency between variance and mean expression values, as compared with more tightly packed spots (MPG vs PT4) reflecting a closer relationship between the data sets. Click here for file

Additional file 3 **Table S2 **Probe sest with expression confined to 4 hr pollen tubes classified according to detection call of the two replicates. Click here for file

Additional file 4 **Table S3 **Complete list of differentially expressed genes between dehisced mature pollen and 4 hr pollen tubes. Click here for file

Additional file 5 **Table S4 **Complete list of differentially expressed genes between leaf and mature pollen grains. Click here for file

Additional file 6 **Table S5 **Complete list of differentially expressed genes between roots/root-hairs and mature pollen grains. Click here for file

Additional file 7 **Table S6 **Complete list of differentially expressed genes between leaf and 4 hr pollen tubes. Click here for file

Additional file 8 **Table S7 **Complete list of differentially expressed genes between roots/root-hairs and 4 hr pollen tubes. Click here for file

Additional file 9 **Table S8**. Complete list of differentially expressed genes between leaf and roots/root-hairs. Click here for file

Additional file 10 **Table S9 **List of genes that showed overlapped expression between 4 hr pollen tubes and root/root-hairs tobacco transcriptomes. Click here for file

Additional file 11 **Table S10 Missing file**. Click here for file

Additional file 12 **Table S11 **A list of tobacco acessions with significant Arabidopsis homologs that had reliable microarray expression in PT4, root/root hairs and overlapped with Arabidopsis root-hair specific transcriptome and virtual proteome. Click here for file

Additional file 13 **Table S12 **List of significant Gene Ontology (GO) annotation of genes that showed overlap expression between PT4 and roots/root-hairs with significant arabidopsis homologs using gProfiler software [83]. Click here for file

Additional file 14 **Table S13 **A list of cell cycle regulators (annotated according to MAPMAN GO terms) with a potential role of maintaining the bicelluar status of the 70% known angiosperms that shed bicellular pollen at anthesis. Click here for file

Additional file 15 **Table S14**. Comparative transcriptome of some putative sperm cell differentiation markers between bicellular and tricellular angiosperms that were induced by Arabidopsis DUO POLLEN 1 [68] and detected in our gametophyte transcriptomes. Click here for file

Additional file 16 **Table S15 **List of primers used in this experiment. Click here for file

Additional file 2 **Table S1 **Mean normalized expression of tobacco probe sets with associated detection calls computed from two biological replicates with expression levels "well above background". Click here for file
