# Supplementary figures and images for: Comprehensive analysis of tobacco pollen transcriptome unveils common pathways in polar cell expansion and underlying heterochronic shift during spermatogenesis
Source: BMC Plant Biol. 2012 Feb 16;12:24. doi: 10.1186/1471-2229-12-24 (PMC3305590; doi:10.1186/1471-2229-12-24)

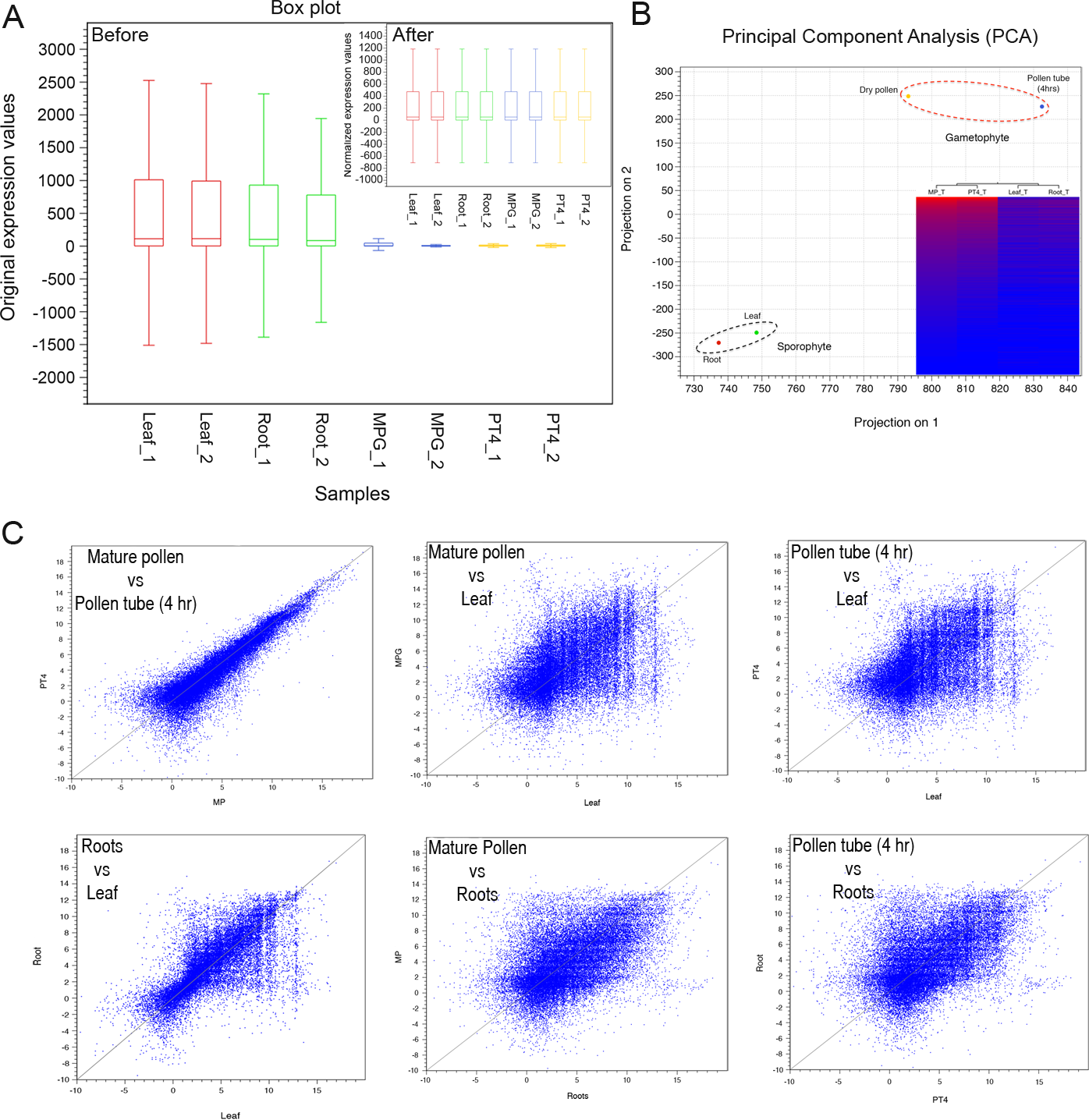

Supplement: Additional file 1 — Figure S1 Data normalization and analysis of replica reproducibility. (a) Box plot of the raw hybridization signal from the four samples, highlighting systematic differences between samples. The inset shows the same data after the normalization. (b) Output of the first and second principal component (PCA) analysis of the log2-transformed data sets. The largest and second-largest principal component (variability projection 1 and 2, respectively) are displayed in orthogonal directions, assessing the overall homogeneity between replicates and variability between samples of different tissue types as reflected in their grouping. The inset shows hierarchical clustering of the same data set grouped according to array similarities and differences. Since both methods produced identical grouping patterns, the quality of the dataset was considered reliable and useful for comparative analysis. (c) Scatterplots of log2-transformed microarray data showing a correlation between tissue arrays. A wider symmetrical scattering of the spots from the 'trendline' implicates higher variability between the tissues, with less dependency between variance and mean expression values, as compared with more tightly packed spots (MPG vs PT4) reflecting a closer relationship between the data sets. [file 1471-2229-12-24-S1.TIFF]
